# Assessment of Changes in Executive Functions and Attention of Cadets as a Result of Military Parachute Jumping

**DOI:** 10.3390/brainsci15010085

**Published:** 2025-01-17

**Authors:** Dariusz Jamro, Marek Kociuba, Maciej Lachowicz, Pawel Korytko, Grzegorz Zurek

**Affiliations:** 1 Department of Physical Education and Sport, General Tadeusz Kosciuszko Military University of Land Forces, 51-147 Wroclaw, Poland; marek.kociuba@awl.edu.pl (M.K.); pawel.korytko@awl.edu.pl (P.K.); 2 Department of Biostructure, Wroclaw University of Health and Sport Sciences, 51-612 Wroclaw, Poland; grzegorz.zurek@awf.wroc.pl (G.Z.)

**Keywords:** executive function, parachute jumping, stress of servicemen, combat readiness, cognitive adaptation, cadets

## Abstract

Objectives: This study analyzed the effects of parachute jump stress on the executive functions and attention of cadets. Executive functions, which includes processes such as attentional control and cognitive flexibility, are crucial for soldiers, especially in situations requiring rapid decision-making. Parachute jumping, as an intense stressor, mobilizes cognitive resources, which can lead to short-term improvements in executive functions. Methods: A total of 64 cadets divided into control (N = 32) and experimental (N = 32) groups participated in the study. The experimental group performed a parachute jump and then took the Color Trails Test, assessed both before and after the jump. Results: The results showed significant improvements in executive functions, in particular, sustained and alternating attention, sequential information processing, and monitoring one’s own behavior, suggesting a positive effect of stress on selected executive functions. Conclusions: The results indicate that intense, short-term stress can positively affect executive functions, although this effect may depend on the type of task and the participants’ experience of exposure to intense stressful stimuli. The study makes an important contribution to the design of future military training, considering the importance of mobilizing cognitive resources in response to short-term stress.

## 1. Introduction

Executive functions (EFs), also referred to as executive control or cognitive control, are a set of elementary cognitive processes that make it possible to process information and respond in ways other than intuitive or learned. They encompass a collective range of cognitive processes such as verbal reasoning, planning, problem solving, resistance to interference or the ability to sustain attention [1]. According to Stuss et al. (1995), level of EFs is reflected by resistance to interference, multitasking, and feedback-based inference [2].

EFs are attributed a particularly important role in dynamically changing or completely new situations in which selective attention, cognitive flexibility, or inhibitory control must be demonstrated [3]. Therefore, these are circumstances in which habitual behavior cannot be relied upon, but require acting in a radically different way from established patterns. In such cases, the success of the task depends largely on EFs, which enable flexible adaptation to unusual situational demands.

EFs also contribute to maintaining cognitive and mental health [4]. They support the maintenance of high levels of physical fitness [5]. The level of EFs is also considered to be an important predictor of school and academic achievement [6,7], and finally they contribute to career development [8] and daily responsibilities [9]. Generalizing the importance of EFs, it can be said that they provide balanced development of cognitive, psychological, and social levels [3].

Attention is a key mechanism in human information processing, enabling the selection of relevant stimuli and the effective ignoring of redundant elements. Research shows that attention not only supports processing, but also acts as a “filter” and amplifier of information to be further analyzed or remembered [10]. Attention guides decision-making processes, reinforcing the neuronal activity associated with the selected options and helps to assign them values, allowing for a more efficient comparison of alternatives [11]. In the case of multi-attribute decisions, attention helps to integrate and evaluate the value of the individual elements to facilitate the final decision [12]. It plays a key role in stabilizing the dynamic process of perceptual and cognitive learning [13]. In the literature, there is a relative consensus that it is difficult to consider attention as a single mechanism; rather, it is a set of cognitive processes that interact with each other and influence other brain processes involved in the performance of cognitive, perceptual, and motor activities. The function of attention is to enable and maintain a specific bodily response in the face of incoming external stimuli [14]. On the other hand, the attentional state is defined as a state of optimal activation that enables the selection of information sources and courses of action. The aim of this mechanism is to optimize the individual’s interaction with the environment according to the meaning of the stimulation or internal intentions and priorities [15].

Military personnel, particularly in command positions, should demonstrate, among other things, a high level of communication ability, which is based on understanding the environment, processing information and outstanding speech competence. A higher level of interpersonal communication competence can be linked to a higher level of EFs, as abilities such as attentional control, emotion regulation, or cognitive flexibility are key to effectively understanding, adapting, and responding to social cues. Individuals with higher levels of EFs can manage their behavior and emotional responses better, which supports the building of positive interpersonal relationships [16,17].

The characteristics of a good leader at the cognitive level are undeniably high perception, the ability to pay selective attention, an above-average ability to assimilate new concepts and information, high levels of working memory and EFs [18,19]. Furthermore, the ability to maintain a high level of attention is extremely important for soldiers. Well-learned actions that are trained on training grounds are automatically triggered by external stimulation, such as environmental, visual or auditory stimuli. These can be the sound of gunfire, the sight of enemy units, changes in terrain, or other combat signals. These stimuli activate learned behavioral patterns, allowing the individual to respond quickly and effectively to combat situations without the need for lengthy decision-making and attention commitment [20,21].

On the other hand, during dynamic test exercises, such as tactical exercises with live ammunition (Force-on-Force), operational simulations in near-real conditions (Live Simulation Training), or training under high stress conditions, the ability to maintain attention becomes crucial [22]. On a changing battlefield, when intentions conflict with automatic responses or require planning a new sequence of actions, planning and implementing appropriate responses and actions consumes a significant amount of attentional resources [23]. For example, in scenarios where unpredictable situations arise, such as a change in mission objective, the need to evacuate, or an emergency, soldiers must focus on the current situation while inhibiting automatic responses that do not fit the new situation [24]. In line with the above, research in the area of cognitive functioning among soldiers takes on particular importance in the context of effectiveness in the execution of planned as well as unexpected training and combat activities.

Researchers from NATO member states draw conclusions and emphasize that EFs for soldiers are a key resource for all aspects of performance, from interpreting the stress factor, cognitive, and physiological processing, to initiating and managing appropriate actions. They also point out that there is an inextricable interdependence between factors that control cognitive functioning and operational performance in dynamic combat situations [25].

An increasing number of studies suggest that acute stress may impair basic EFs such as working memory, attentional control, inhibition, and cognitive flexibility. An example of an activity that induces extreme stress is parachute jumping (PJ). It induces numerous physiological responses that have been studied in a variety of scientific contexts. In response to the intense mental and physical stimulation of PJ, the body responds by activating the hypothalamic-pituitary-adrenal (HPA) axis, leading to an increase in cortisol levels [26]. In addition, increases in adrenaline, noradrenaline and heart rate have been observed, indicating a strong stimulation of the sympathetic nervous system during parachute jumping (PJ) [27]. For some participants performing a PJ, changes in cognitive functions, such as visual-spatial abilities, and hormonal responses associated with repeated exposure to the stress associated with the jump have also been studied [28]. Analysis of these responses provides valuable information about the body’s adaptive defense mechanisms in the face of extreme conditions.

Studies by Messina et al. (2016) and Deinzer et al. (1997) demonstrated that PJ is accompanied by a dissociation of sympathetic nervous system and HPA axis response patterns, showing a slower recovery of sympathetic activity than cortisol secretion. The above study also showed that cortisol, prolactin (PRL), and growth hormone (GH) levels increase dramatically in response to PJ stress in individuals preparing for a PJ. The study involved young men (22 to 36 years old) performing their first PJ. Immediately prior to the PJ, an almost twofold increase in cortisol, adrenaline, and noradrenaline levels was observed compared to the value recorded after the PJ, indicating a strong stress response of the body to the PJ. These reactions were most intense for the first PJ, and decreased in intensity as the task was repeated (e.g., during the second and third PJs), reflecting the adaptive nature of the body’s response to repetitive stress [29,30]. Additionally, distinct stress responses, such as increases in cortisol, heart rate, sympathetic dominance, and anxiety were noted both during the immediate anticipation of the PJ and up to 15 min after the jump. In contrast to these hormones, testosterone levels remained low throughout the study period, suggesting a specific endocrine system response to life-threatening stress [30,31].

A study by Taverniers et al. (2011) analyzed reactions to the first PJ among cadets. The aim was to investigate the effects of extreme stress on salivary cortisol levels and cognitive abilities, in particular visual-spatial learning. Participants performed a task involving following a path with multiple repetitions to assess their ability to remember the route and make decisions related to spatial processing. The results of the study confirmed that PJ induces a significant increase in salivary cortisol levels and a subjective feeling of increased stress. In addition, a significant impairment of visual-spatial learning ability was observed after exposure to stress. The more complex the task, the greater the cognitive impairment. This indicates that extreme stress induced by PJ can negatively affect the functioning of the hippocampus and retrosplenial region, which are responsible for spatial processing. The study provided evidence of how intense stress can impair cognitive performance, particularly in the context of tasks requiring spatial information processing [28].

It has been proven that PJ has no significant effect on HR, HRV, stress hormones at night or on orthostatic test results on the morning of the PJ. A study by Hynynen et al. (2009) suggested that a highly stressful event in the form of a PJ had no anticipatory effect on cardiac autonomic modulation. Such results may indicate that the autonomic response to extreme stress is stimulus-dependent rather than related to its anticipation. This mechanism may be explained by a delay in the activation of the HPA axis and the sympathetic nervous system, which prepare the body for immediate action in the face of danger, rather than eliciting a response to the anticipatory imagery of the stressor itself. In contrast, both novice and experienced parachutists had significantly emphasized sympathetic activation during the PJ [27].

The effects of acute stress, including stress induced by military exercises, on basic EFs remain unclear in the literature. The discrepancies concern both the neurobiological mechanisms underlying these processes and the direction and intensity of the stress effects. This leads to uncertainty about how—or if at all—high-intensity stress affects abilities such as attentional control, cognitive flexibility, or decision-making.

In their meta-analysis on the effects of acute stress on EF, Shields et al. (2016) analyzed 33 studies on the effects of stress on working memory, inhibition, and cognitive flexibility. The results showed that stress significantly impaired working memory and cognitive flexibility, limiting the ability of individuals to adapt when presented with new information and changing situations. In contrast, the effect of stress on inhibitory control was more complex and task-specific. In some cases, stress improved inhibition, especially when it required suppression of dominant responses, but in other tasks, stress led to impaired performance. The authors of the aforementioned studies emphasize that the effect of stress on inhibition may depend on a variety of factors, such as the level of emotional arousal and the context of the task. The above meta-analysis also indicated that acute stress may improve selective attention, suggesting that EFs related to ignoring irrelevant stimuli may improve in high arousal situations [32].

Research on the effects of stress on attention indicates that stress may improve selective attention, particularly by reducing consideration of stimuli irrelevant to the task at hand. This observation is consistent with the selective attention model, which predicts that stress mobilizes cognitive resources, resulting in more focused attentional control on key aspects of the task. For example, during a PJ, the parachutist is in a state of full concentration on the procedures and activities required during the jump, as they directly affect his or her safety and the lives of the other participants involved in the jump. In such a context, stress contributes to an increased focus on activities critical to survival, ignoring less important stimuli [33].

There is no doubt that PJ presents a significant and immediate psychological and physical challenge to the novice parachutist. Young cadets may feel the excitement and euphoria of taking on the new challenge of PJ. On the other hand, they are also accompanied by fear of the potential risk of injury or even death, which can significantly affect their stress levels and physiological arousal. After all, there is always the risk of complications, failure of the parachute to open, technical failure, or human error. Many parachutists similarly report that they do not remember much or even anything about their first PJ since jumping out of the aircraft. Some cadets may experience denial, which, in a specific training context such as PJ, is an adaptive stress response. Under extreme conditions, this denial may have a protective function, minimizing the experience of anxiety and allowing the task to be completed. However, taken out of this context, such behavior could suggest pathological defense mechanisms. As reported in the literature, but especially by observations on the ground, almost all novice parachutists show some signs of cognitive impairment [34,35].

Furthermore, military parachuting research can combine intensive, realistic exercise with controlled experimental conditions, which is usually difficult to achieve outside the laboratory. Such research allows observation of how real-world, stressful combat situations affect soldiers’ cognitive abilities, while allowing researchers to precisely monitor and control variables, increasing the reliability of the results.

Results from previous studies (e.g., Shields et al. (2017), Qi et al. (2018)) suggest a possible significant effect of high but short-term stress on attention and related EFs [32,36]. For this reason, the study hypothesized that levels of attentional performance and executive functioning in selected areas would improve immediately after the extreme stress stimulation of performing a first PJ with a steerable and gliding parachute. The aim of the experiment was to investigate whether (and if so, how) executive functioning and attentional performance would undergo changes as a result of a potentially life-threatening military exercise, i.e., a first PJ using a group “B” parachute, a steerable and gliding parachute with a combined opening system (with stabilization) among a group of cadets.

## 2. Materials and Methods

### 2.1. Participants

The group under study were cadets in their third year of a long-cycle Master’s degree program with the major in command, studying at the General Tadeusz Kosciuszko Military University of Land Forces in Wroclaw with the military occupational specialty called general reconnaissance and airborne forces. A group of cadets specializing in general reconnaissance and trained as airborne forces were deliberately selected for study in the context of PJ and combat training, as both specializations require specific skills related to operations in extreme conditions and rapid adaptation to changing tactical conditions. PJ, as a component of training, is a key aspect of soldiers’ preparation, as it allows rapid movement to hard-to-reach areas that may be crucial for reconnaissance operations. In addition, they are an integral part of the training of airborne combat units, which must rapidly adapt to changing operational situations on the ground.

All participants studying these specialties were included, but due to the small number of women studying these specialties, only the results obtained by men were analyzed in the study (N = 64, mean age 22.94 years, SD = 1.26). From the selected group of participants, simple random sampling was performed [37,38], in which each person had an equal chance of qualifying for one of the groups. Using a random number generator, an experimental group (EG) (N = 32, mean age 22.79 ± 0.49, (22.30–23.27) years) was selected to perform a PJ, and the remainder of the participants formed a control group (CG) (N = 32, mean age 23.07 ± 0.42, (22.66–23.49) years). Furthermore, a condition for inclusion in the study was that participants in both groups had exactly the same parachuting experience. Both the EG and CG had completed a basic PJ course two years prior to the experiment, which consisted of five PJs with a group “A” parachute. The group “B” parachute, compared to the group “A” parachute, differs primarily in that it is more steerable and allows free choice of landing location. At the same time, the prerequisite for being allowed to jump on gliding and steerable parachutes is successful completion of the group “A” parachute course.

Participants in the study gave informed consent to voluntarily take part in the study and were also examined by a doctor, who took a full medical history and conducted a physical examination. Prior to PJ, participants in both the EG and control CG completed an obligatory ten-day parachute training course preparing them to perform their first PJ using group “B” parachutes and performed motor fitness tests allowing them to perform PJs in accordance with regulations and procedures in the Polish Armed Forces. The test was conducted at the Airborne Forces Training Centre of the 25th Air Cavalry Brigade in Leznica Wielka (Poland).

### 2.2. Procedures

On the first day of the study, both the EG and the CG took the first CTT version “A” 24 h before the EF performed the PJ. The participants in the CG were not aware during the first CTT test that they would not participate in the PJ scheduled for the following day. At the first stage of the study, none of the participants knew to which research group they belonged.

**Figure 1:**
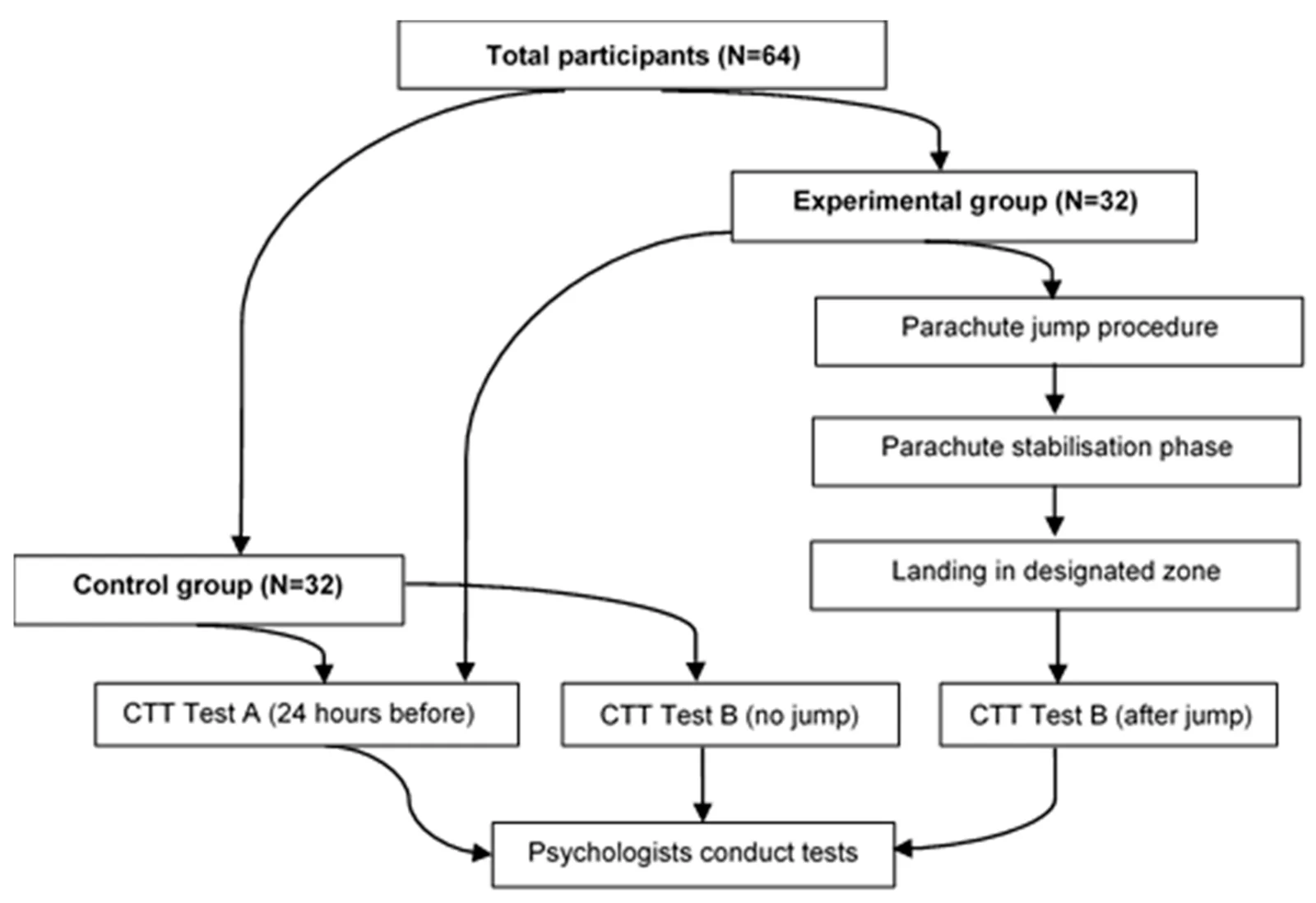
Diagram of the organization of the study. Division of participants (N = 64) into experimental (N = 32) and control (N = 32) groups, with description of test procedures and parachute jump phases.

The testing of EFs both before and after the jump was carried out by two psychologists under the same conditions, i.e., at specially prepared stations in the drop zone (tent, two chairs, table, pencil, stopwatch and CTT-1 and CTT-2 sheet). Figure 1 shows a diagram of the organization of the study, illustrating the division of participants into groups (control and experimental) and the sequence of test activities and procedures.

**Figure 2:**
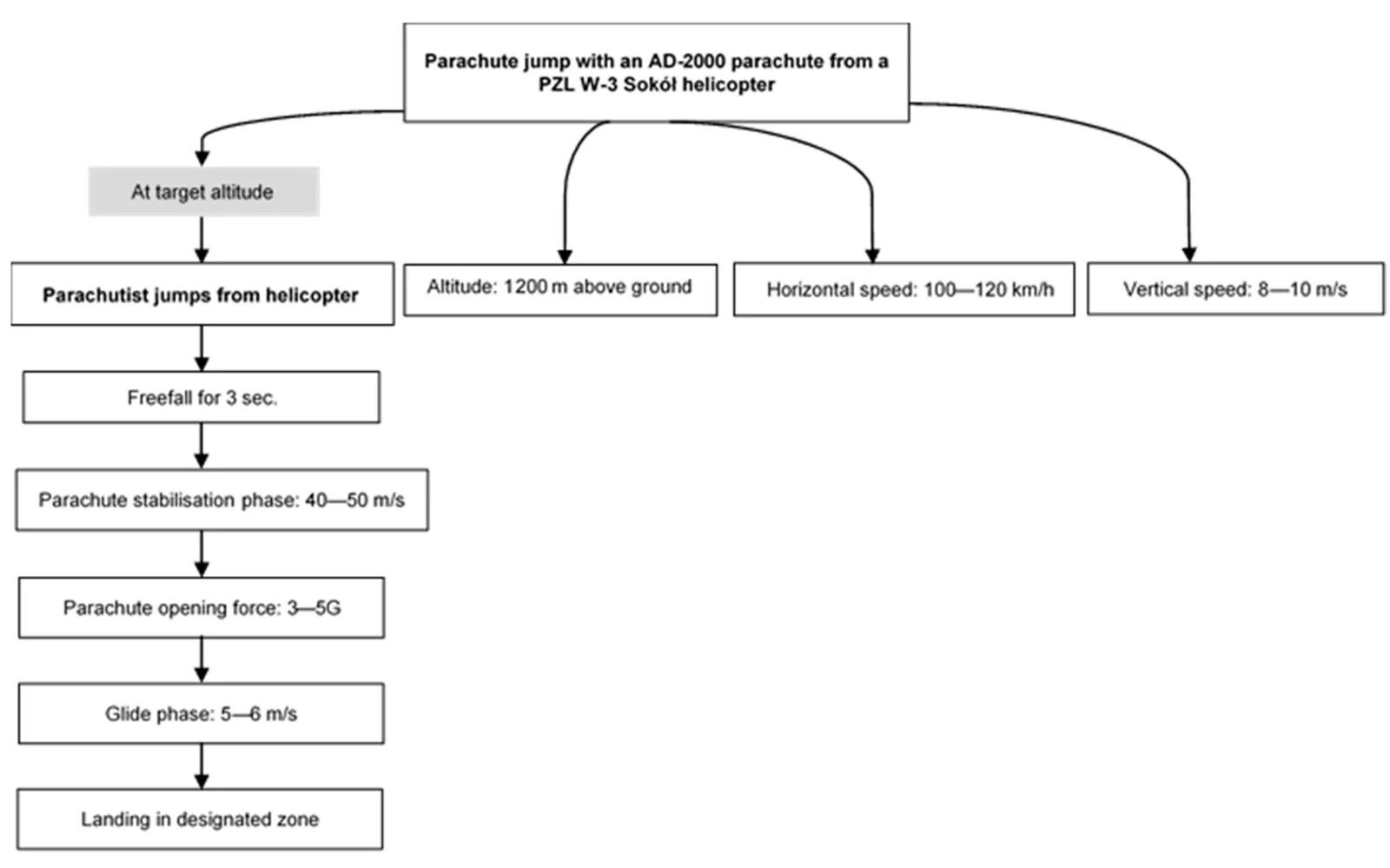
Detailed procedure of parachute jump with an AD-2000 parachute from a PZL W-3 Sokół helicopter.

The following day, cadets from the EG performed their first PJs. These were jumps using a group “B” steerable and gliding parachute equipped with a combined opening system (with stabilization), model AD-2000. Prior to the PJs, each test participant underwent a detailed preparation. This included an equipment check, including a thorough inspection of the AD-2000 parachute. Participants also underwent a mandatory briefing, during which emergency procedures, jump conditions, and wind direction were discussed. All preparatory procedures took place in accordance with current safety standards. The jumps took place from aboard a PZL W-3 Sokół helicopter. At the time of the drop, the helicopter was flying at a horizontal speed of between 100 and 120 km/h, with a rate of climb of around 8–10 m/s. The jumps were conducted from an altitude of 1200 m above ground level. After reaching the planned altitude, each participant jumped out of the helicopter on the command of the jump supervisor, proceeding directly to the freefall phase. Within the first three seconds of the jump, the AD-2000 parachute stabilization system activated automatically to keep the parachutist’s position optimally stable. The fall speed during stabilization was approximately 40–50 m/s (144–180 km/h), depending on the weight of the parachutist and weather conditions. After the stabilization period, the AD-2000 parachute opened automatically using a combined opening system. At the moment of parachute opening, the parachutists were subjected to an overload force of 3–5 G. Once the canopy was fully open, the parachutists entered the glide phase, during which they could control the direction and speed of their flight using the AD-2000 parachute controls. The speed of descent with the parachute was about 5–6 m/s (18–21 km/h) (Figure 2). The glide phase lasted from a few to several minutes, depending on the height of the canopy opening and the prevailing weather conditions. Each parachutist landed in a designated zone. During the landing, the parachutists used a technique of decelerating their descent speed, which allowed them to complete the jump in a safe and controlled manner.

Parachutists from the EG, after landing in the designated area on the drop zone, proceeded to the post-landing assembly point, where immediately after the jump, they completed the CTT again; this time, the “B” version. The test was taken by all participants during the fourth minute after landing. Participants from the CG also completed the test again at this time; however, it was not preceded by a PJ. Immediately prior to the second stage of the study, each participant was informed whether they would be performing the parachute jump or not.

### 2.3. Color Trails Test (CTT)

A comprehensive study of the level of EF and attention is a difficult, complex, and time-consuming process. Cognitive functioning is measured using test tools, neuroimaging methods or dichotic tests. Although today, advanced tools for accurate, real-time testing of cognitive function, such as brain mapping and neurophysiological methods, among others, can be used, their cost and scarcity of availability significantly limit their range of application [39,40].

In order to assess the level of attention and EF, a simple and cost-effective method, namely the CTT, was used in the study. The chosen test was selected for its ease of use and feasibility of conducting it in the field. The CTT is widely used in studies of cognitive function in healthy individuals and in the diagnosis of neuropsychological disorders such as dementia, brain injury or psychiatric disorders. It is also used in studies related to stress, cognitive load, or the effects of various factors (e.g., medication, exercise, or extreme experiences) on cognitive performance [41]. The CTT is one of the most popular neuropsychological tests for assessing the level of cognitive processes, specifically assessing sustained and alternating attention, sequential information processing, monitoring of one’s own behavior, and intentional search for material [42,43].

### 2.4. Structure and Conduct of the CTT

The CTT consists of two parts: CTT-1 and CTT-2. Each of these parts measures different aspects of EF and attention. The subjects first performed the CTT-1 test and then the CTT-2. In the CTT-1 test, the participant was given a card containing 25 circles numbered from 1 to 25, which were randomly distributed on an A4-sized page. Each circle was filled in with one of two colors (yellow or pink). The task was to connect the dots in numerical order (from 1 to 25) regardless of color. This part of the test primarily assessed selective attention and speed of information processing. In the CTT-2 test, the participant was given a second A4 sheet containing 49 circles, numbered from 1 to 25. The participants’ task was to connect the dots in numerical order, except that they had to choose alternating circles of different colors (e.g., from pink to yellow). In addition, every number except the number 1 appeared twice in circles of different colors. The CTT-2 was the more demanding part of the test, as it required simultaneous cognitive control, cognitive flexibility, and the ability to inhibit automatic responses. It also assessed EFs such as planning, response inhibition, and switching between different sets of stimuli [43].

### 2.5. Application and Interpretation of Results and Key Variable

The test score was expressed as the time needed to complete each part. Longer completion times indicated difficulties in information processing, EFs, or attention. CTT is independent of language skills, allowing it to be applied to different cultural and linguistic groups. The ease of administration and the relatively short time required to perform the test (usually less than 10 min) make it a convenient diagnostic tool [43].

### 2.6. Statistical Analysis

In order to assess the normality of the distribution of the variables, the Shapiro–Wilk test was used (all variables had a normal distribution). Mean values, standard deviations, coefficients of variation, and 95% confidence intervals were calculated. The level of statistical significance in the analysis was set at *p* < 0.05.

In order to compare the significance of differences between the EG and the CG in mean CTT-1 and CTT-2 results before the PJ (in the first test) and after the PJ (in the second test), the Student’s *t*-test for independent samples was used.

For the analysis of statistical significance of intra-group differences in mean CTT-1 and CTT-2 scores in the CG and EG, a Student’s *t*-test for dependent samples was used. The analysis included a comparison of results before and after the PJ in the EG and a comparison of results between the first and second tests in the CG.

The study was based on the approval of the Commission for the Ethics of Scientific Research of the Wroclaw University of Health and Sport Sciences (No. 2/2021 of 2 December 2021). All research procedures involving human subjects were conducted in accordance with the 1964 Declaration of Helsinki and its subsequent amendments or other comparable ethical standards. The study was conducted as part of a scientific project entitled “Physical fitness and cognitive functioning vs. combat training of cadets of the General Tadeusz Kosciuszko Military University of Land Forces in Wroclaw” (project No: 39/WNB/17/SMON).

Statistical analyses were conducted at the Biostructure Research Laboratory of the Wroclaw University of Health and Sport Sciences using IBM SPSS Statistics 27 software (https://www.ibm.com/products/spss-statistics, retrieved: 15 September 2024). The laboratory is ISO 9001 [44] certified, which guarantees compliance with high quality standards.

## 3. Results

Table 1 shows the basic descriptive statistics of the results of the analyzed variables and the analysis of the differences between the groups: the CG, which did not perform a PJ, and the EG, which did. The above results show that there were no significant statistical differences between the EG and the CG in all the CTT analyzed (completion time) both before (first test) and after the PJ (second test). The *p*-values in all cases significantly exceed the 0.05 significance threshold, confirming that the differences between the CG and the EG did not reach statistical significance in any case.

For the results of the CTT-1 ver. A test before the PJ for the EG and in the first study for the CG, it can be observed that the mean time to complete the CTT in the EG was longer than in the CG; the standard deviation in the EG (12.30) additionally indicated greater variability in this group, meaning that the participants’ performance was more variable compared to the CG. On the other hand, the confidence interval for the EG, although wider than that for the CG, suggests that with 95% probability the true mean of this variable is between the values of 35.10 and 43.97. The wide interval may be due to the greater variability in the results in the EG.

Analyzing the CTT-1 results after the PJ in the EG and in the second test in the CG, the mean CTT completion time in the EG was slightly shorter than in the CG. Similar to the CTT-1 results, the standard deviation was higher in the EG (12.45 vs. 8.99), indicating a greater dispersion of results compared to the CG. The confidence interval for the EG was wider than for the CG, reflecting greater uncertainty about the true mean value.

When analyzing the results (mean time to complete) of the CTT-2 test before performing a PJ in the EG and in the CG, it is noteworthy that the value of this variable for the EG was significantly higher than in the CG, indicating an average longer time needed to complete the CTT-2 test for participants in the EG compared to the CG. However, there were no statistically significant differences between the groups in this variable. The higher standard deviation in the EG compared to the CG (16.56 vs. 10.53) further indicated a higher variability in results in this group. The width of the confidence interval for the EG was also significant, suggesting greater uncertainty regarding the precise estimation of the mean.

When analyzing the results (mean completion time) of the CTT-2 test after a PJ in the EG and the CG, the values of this variable were most similar between the groups. The mean CTT completion time in the EG was only slightly shorter than in the CG. Both standard deviations were similar (15.94 vs. 15.23), as were the confidence intervals. The value of the confidence intervals indicated that there is a 95% probability that the true means fall within fairly close ranges.

Table 2 shows the results of the statistical analysis for the CTT-1 and CTT-2 tests, comparing the results before and after the PJ in the EG and comparing the results of the CTT-1 and CTT-2 tests between the first and second tests in the CG. Statistically significant results (*p* < 0.05) are indicated in bold. The main results of the present study are the results of the repeated statistical analysis, which showed significant differences in the mean time to perform CTT-1 before the PJ compared to the mean time achieved after the PJ (*p* = 0.010), and also revealed a significant difference in the mean completion time of CTT-2 before the PJ compared to the mean completion time of CTT-2 after the PJ (*p* = 0.018) in the EG. The cadets in the EG achieved significantly shorter (better) times immediately after the PJ in both the CTT-1 test (improvement from an average time of 39.53 s to 34.19 s, a shorter average time by 5.34 s) and the CTT-2 test (improvement from an average time of 72.59 s to 64.69 s, a shorter average time by 7.9 s).

**Figure 3:**
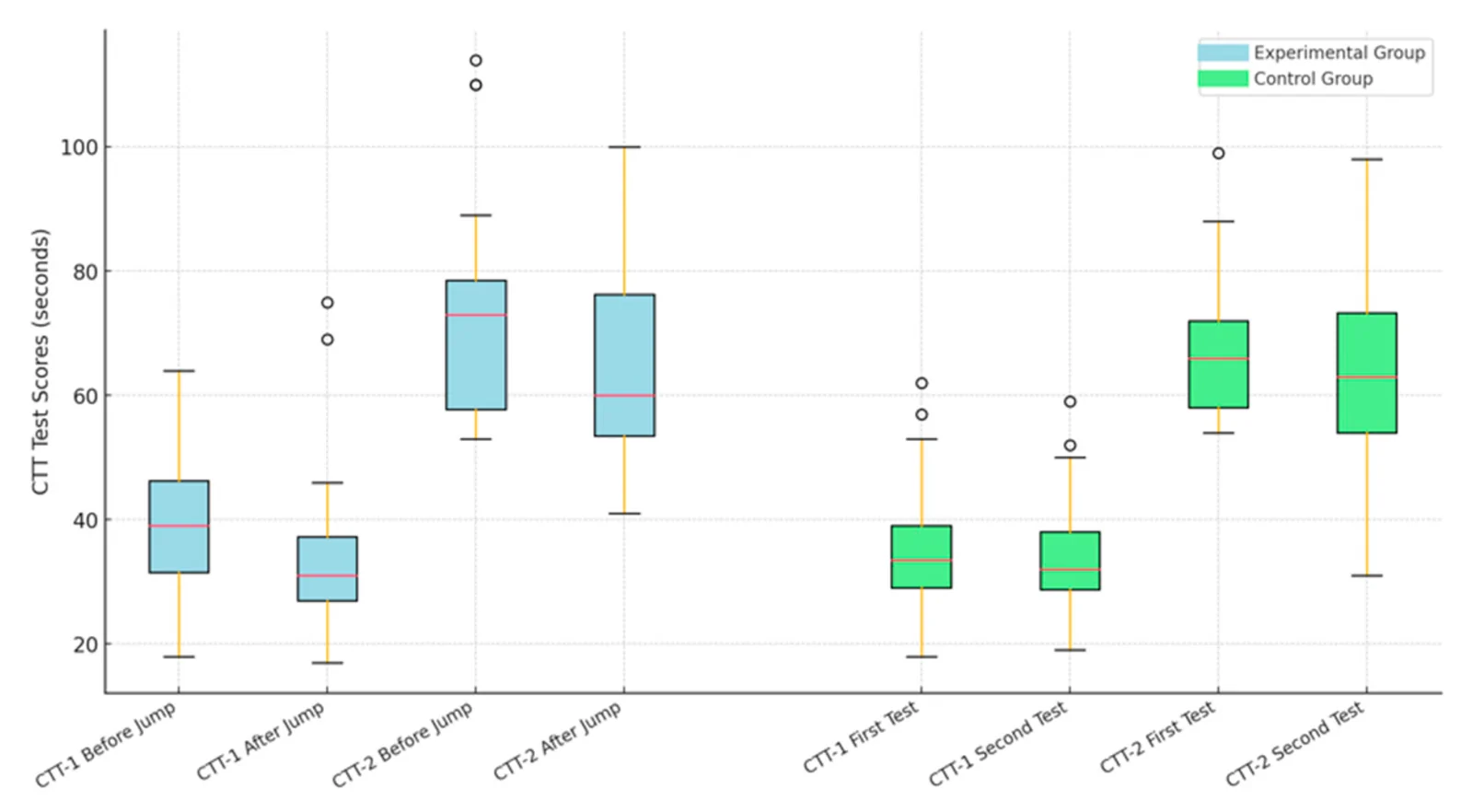
CTT test scores before and after the parachute jump in the experimental group and in the first and second tests in the control group.

For the CG, the analysis showed no statistically significant results, indicating that there were no significant changes between the first and second CTT-1 and CTT-2 test results. Figure 3 shows a graphical representation of the mean CTT-1 and CTT-2 test scores in the EG before and after the PJ and a graphical representation of the mean CTT-1 and CTT-2 test scores in the CG for the first and second test. The horizontal axis (x) shows the different test conditions: CTT-1 (1), results in CTT-1 (version A) before the PJ in the EG and for the first test in the CG; CTT-1 (2), results in CTT-1 (version B) after the PJ in the EG and for the second test in the CG; CTT-2 (1), results in CTT-2 (version A) before the PJ in the EG and for the first test in the CG; and CTT-2 (2), results in CTT-2 (version B) after the PJ in the EG and for the second test in the CG. The vertical axis (y) shows the average times (in seconds) achieved in each of these conditions. The yellow color indicates the EG that performed the PJ. The orange color indicates the CG that did not take part in the PJ. Before the PJ (CTT-1 (1) and CTT-2 (1)), the EG obtained higher mean times in the CTT than the CG. After the PJ (CTT-1 (2) and CTT-2 (2)), the differences in mean times between the EG and CG appeared smaller, especially for CTT-2 (2), where the values were almost equal (64.69 s for the experimental and 65.12 s for the control). The above differences between the groups both before the PJ (in the first test) and after the jump (in the second test) were not statistically significant (Table 1).

## 4. Discussion

The results of the present study revealed that performing a PJ, as an extremely stressful experience, can lead to improvements in EF and attention. Study participants in the EG performed significantly better on the CTT immediately after the jump than before the jump. The significant reduction in test completion time in both the CTT-1 and CTT-2 versions of the test may indicate a mobilization of cognitive resources and an improvement in alternating attention and the ability to sequentially process information and purposefully search through material as a result of intense stressful arousal [32].

The results of our study supported the hypothesis that the performance of attention and EFs in selected areas improves immediately after the extreme stress stimulation of performing the first jump with a steerable and gliding parachute. In the context of the available literature, these results fit with the theory of an increase in attentional selectivity under stress and may suggest the existence of adaptive mechanisms activated in response to severe but short-term stress [32,33]. However, when compared to other studies with similar subjects, differences can be seen in both the methodology and the results, which in some cases suggest that the picture is more complex. Studies on stress and attention may differ in the way the experiments are conducted, e.g., in the type of stress induced (emotional, physical), the intensity of the stress, the way attention is measured, or in the study populations (age, baseline stress level). Such differences can affect the results obtained, leading to difficulties in directly comparing studies. Furthermore, the effect of stress on attention is ambiguous and depends on a number of factors, such as the duration of stress (short-term vs. chronic), the type of task involving attention, or individual differences between the subjects. For example, stress may not only increase attentional selectivity, but also lead to a deterioration of the overall ability to focus or to more dynamic changes in attention, depending on the situation [26,28].

Referring to the comparative literature, a study by Taverniers et al. (2011) showed an analysis of the effects of PJ on cognitive function, measuring the cortisol response and visual-spatial abilities of cadets. As in our study, the participants were cadets and the sample size was almost the same (N = 61), while the cognitive tasks included more complex visual-spatial tests. Participants performed a visuo-spatial path learning task to assess their spatial-visual memory and information processing abilities. Taverniers et al. observed a significant deterioration in performance in visual-spatial tasks immediately after exposure to a PJ. These results differ from ours, as our study reported an improvement in CTT scores after the jump. This may be due to differences in the nature of the tests; CTT tasks are less complex and require less visual-spatial engagement, focusing more on sequential and alternating attention. At the same time, Taverniers et al. suggested that less complex tasks such as the CTT compared to visuo-spatial path learning tasks may be less susceptible to the negative effects of high stress, which may explain the lack of deterioration in CTT scores in our study and, on the contrary, their significant improvement. The differences in PJ experience between the study groups should also be highlighted. The cadets from our study, both from the EG and CG, had already made five jumps in the past (about 2 years before the study) as part of a basic course, but on a different type of parachute, whereas the cadets from the study by Taverniers et al. had made their first PJ in their lives [28].

The parachutists in the above study by Taverniers et al. with no PJ experience were likely to have been exposed to significantly higher levels of stress, which may have translated into poorer performance in visual-spatial tasks. In contrast, for the cadets in our study, the stress associated with a first PJ with a more advanced type of parachute may have played a mobilizing role, which translated into more effective use of cognitive resources. This manifested as improved selective and alternating attention, resulting in significantly better performance on the CTT after the jump. Thus, the stress experienced by the cadets appeared to have a supportive rather than crippling effect, supporting their cognitive abilities in a situation requiring high levels of concentration and adaptation.

In addition, the cadets of the reconnaissance and airborne forces specialty taking part in our study had previous experience in operating in demanding and stressful conditions, which may have significantly influenced their response to the stress stimulus of their first PJ on a gliding and steerable parachute. Previous experience with activities such as mountain climbing, intensive field and tactical training, participation in a SERE (Survival, Evasion, Resistance, and Escape) course, tactical training on skis, and previous PJs on a less steerable parachute may have prepared the participants for stress management. Such experience with exposure to stress allows the development of coping mechanisms, which reduces reactivity to stressful stimuli and strengthens cognitive processes in highly stressful situations [19]. Therefore, the cadets were able to better control their physiological and cognitive response to stress when embarking on a PJ, allowing for the mobilization of cognitive resources and a short-term improvement in EFs, including attentional selectivity, without experiencing a crippling effect.

Another comparative study is a meta-analysis by Shields et al. (2016), which examined the effects of acute stress on key EFs: working memory, inhibitory control, and cognitive flexibility. The results indicated a negative effect of stress on working memory and cognitive flexibility, which differs from the observations in our study, where improvements in CTT were noted after PJ. A key difference between our own study and the work of Shields et al. is the range of functions assessed. Our own study focused on basic EF skills such as sequential information processing, behavioral monitoring, and purposeful searching of the content, which may be less susceptible to the negative effects of stress. At the same time, Shields et al. emphasized that the impact of stress depends on the type of task and the intensity of the stressor. Their analysis included studies in which stress was induced in the laboratory using a variety of methods, including arithmetic tasks under time pressure, situations that induce uncertainty, noise, or social stressors. EFs were assessed using standardized tests such as the Stroop Test (cognitive inhibition), Go/No-Go (response inhibition), Digit Span (working memory), and task-switching tasks (cognitive flexibility). However, the results from our study are partly consistent with the reports of Shields et al., as it was pointed out that the effect of acute stress on inhibitory function is not always conclusive; some studies reported improvements in response inhibition. Elevated levels of stress-related arousal probably promoted the suppression of automatic responses, improving performance in tasks requiring selective attention, similar to the CTT from our study. Factors supporting the improvement may also have been the short duration of stress, an adequate level of stress that did not exceed a threshold causing cognitive disorganization, and the participants’ motivation to perform well on tasks [32].

An alternative comparative study that should be noted is the work of Friedl et al. (2016), which analyzed the stress response in soldiers under simulated and combat conditions, showing that realistic combat exercises have a more complex effect on EF levels than laboratory stress tests. In our study, the experiment was conducted under authentic conditions of parachuting, which brings it closer to real battlefield stress situations than typical laboratory tests. The effect of stress on the mobilization of cognitive resources under authentic combat conditions may differ from laboratory conditions. The effect of mobilization under stress may be more intense in conditions that are closer to real operations, which is consistent with our results [25].

The results of our study, showing improved performance on the CTT after PJ, are consistent with the model developed by Arnsten (2009). According to this model, brief and intense stress activates specific signaling pathways in the prefrontal cortex (PFC), such as noradrenergic and dopaminergic pathways, leading to short-term improvements in EFs, including attentional selectivity and inhibitory control. Under conditions of acute stress, norepinephrine and dopamine levels increase, which may improve focus and alternating attention through increased PFC activation. At the same time, Arnsten’s model assumes that prolonged exposure to stress weakens PFC structures, leading to depletion of cognitive resources and reduced EF skills [45]. The CTT, as a task of moderate difficulty, may have been a task suitable for activating the aforementioned mechanisms, which promoted better performance on the task without leading to depletion of cognitive resources.

Another indirect comparative study worth mentioning is the work of Deinzer et al. (1997), who analyzed hormonal responses to multiple PJs and showed that cortisol levels were highest on the first jump and then decreased on subsequent jumps. This result suggests that the hormonal response to stress, as measured by cortisol levels, becomes habituated with repeated exposures to the same stressor, indicating adaptation of the body to stress [30]. In our study, the cadets, although they had previously undergone basic PJ training, made their first jump on a new, more advanced parachute, which may have restored the novelty effect of the stressor and triggered a strong hormonal response and an increase in psychophysiological arousal, leading to improved performance on executive tests.

A recent study by Fu et al. (2024) showed that intense exposure to repeated stressors, such as PJ, can lead to impaired inhibitory control and other EFs as a result of repetitive subconcussions and chronic stress [46]. Participants in this study were more experienced and subjected to multiple exposures to the stressor, which contrasts with our study in which participants performed a single PJ. The results of a study by Fu et al. suggest that prolonged and intense exposure to stressors may lead to an overload of cognitive resources and negatively affect inhibitory control. Our results, indicating improvements in the CTT, suggest that a single, intense stressor may have the opposite effect, mobilizing cognitive resources without causing overload. These differences highlight that the effect of stress on EFs depends on the intensity and repetition of the exposure.

From a theoretical perspective, our results make an important contribution to the understanding of how intense, short-term stress affects the mobilization of cognitive resources. Most notably, our study supports the theory that stress can act as a reinforcing factor in the context of EFs, particularly selective attention. In his study of selective attention and performance in hazardous environments, Baddeley (1972) showed that stress can positively affect the ability to focus attention on key stimuli, which is particularly important in contexts requiring rapid response. Baddeley’s research suggests that under conditions of intense but brief stress, attention becomes more selective, enhancing cognitive performance [47].

In addition, our results are in line with research carried out by McEwen (2016), who points out that while short-term stress can mobilize cognitive resources and provide benefits such as improved EF skills, long-term exposure to stress leads to negative changes in brain structures, including the hippocampus and prefrontal cortex. McEwen emphasizes that chronic stress leads to neuronal damage and impaired memory function and emotional regulation, resulting from a stress overload. Our study, which demonstrated the positive effects of a single, intense stressor, confirms that the beneficial effects are usually short-lived and result from the mobilization of cognitive resources. However, as McEwen points out, prolonged exposure to stress can lead to depletion of these resources and induce long-term, destructive changes in the nervous system [48]. Hence, it is crucial that results such as those obtained in our study, associated with short-term mobilization of cognitive resources, should not be interpreted as evidence of the positive effects of long-term stress. A short-term stressor such as a PJ may temporarily improve EF and selective attention, but prolonged or repeated exposure to stress may overload the cognitive system and lead to negative effects.

The results of the present study may have important applications in military training programs, especially those that are regularly exposed to combat stress. PJ, being one of the most stressful military exercises, has proven to be an effective tool for shaping EFs and adaptive skills in combat situations. Results indicate that short-term, intense stress can contribute to improvements in selective attention and the ability to process key information quickly, which is essential in combat settings requiring rapid decision-making [33]. In addition, the use of PJ simulation as part of training can contribute to training effectiveness in other tasks requiring rapid adaptation, such as reconnaissance operations or rescue missions [25].

At the operational level, the results suggest that introducing controlled stress during training can serve as a form of psychological training, allowing soldiers to be better prepared for combat situations. In a training setting where the development of reaction speed and precision is a priority, scheduling a sequence of stress-intensifying exercises can help shape cognitive resilience, which is crucial for soldiers operating on the battlefield [29]. In the future, it would be worth considering the use of this method also in other special units that operate in extreme conditions, such as special forces, which may be exposed to rapid situational changes and require a high level of adaptability [21].

Another practical implication is that the results can be applied to the development of training programs that will systematically enhance participants’ cognitive control and adaptability under stressful conditions. Research indicates that the experience of a combat situation can lead to short-term improvements in EF, particularly in situations requiring the suppression of automatic responses [32]. The effectiveness of this mechanism can be enhanced by incorporating stress regulation techniques, such as mindfulness training, which enables soldiers to more consciously control their emotions and concentration [49]. Such an approach can have tangible benefits by enabling military units to better respond to dynamically changing operational environments [23]. Finally, from a military psychology perspective, the results may provide guidance for professionals working with soldiers on how to design training to minimize long-term stress and support cognitive function to better prepare for combat tasks.

## 5. Limitations

A limitation of the study is its focus on neuropsychological indicators only, whereas studies carried out by, e.g., Hare et al. (2013) analyze hormonal indicators of stress and show significant differences between groups with different levels of experience [26]. Future studies could be extended to include hormonal and psychophysiological indicators, such as cortisol levels or heart rate variability, to provide a more comprehensive picture of the stress response in the context of cognitive functioning [50].

Another issue is the selection of the CG. In our study, the CG consisted of cadets who had not taken a PJ, but who had gone through the same test procedure and had the same PJ experience. Some previous studies have included participants who were completely unrelated to the PJ context as a CG which may allow for a wider generalization of the results [51]. However, the inclusion of military cadets in our CG allowed us to minimize the impact of demographic differences and the specific military context on the results.

Finally, EF is a complex cognitive function that includes several components. While this study assessed certain aspects of EF, it is important to emphasize that only specific components were tested (using CTT), such as cognitive flexibility and attention-shifting. Consequently, the findings cannot be generalized to all components of EF. Future studies should employ a wider range of EF assessments, including tests of working memory, inhibitory control, and decision-making, to ensure a more comprehensive understanding of EF and its response to stressors.

## 6. Conclusions

In conclusion, the results of our study indicate that intense, short-term stress, such as that experienced during a PJ, can positively affect EF and selective attention. This effect may be due to the mobilization of cognitive resources in response to stress. In the context of combat conditions, where rapid and efficient mobilization of cognitive resources is crucial, such short-term stressors may support cognitive performance. In addition, short-term stressors can serve as a useful practice tool in military training, helping to prepare soldiers to operate in situations requiring rapid adaptation and decision-making.

Supervised and guided exposure of soldiers to stressful situations can support the development of their EFs, such as reaction speed and selective attention, which is particularly important in dynamically changing operational environments.

## Figures and Tables

**Table 1:** Descriptive statistics of the analyzed variables in the EG and CG.

Variable	Statistics	Group		Student’s *t*-Test (EG vs. CG)		
		EG (N = 32)	CG (N = 32)	t-Statistic	*p*-Value	df
Before the jump CTT-1 “ver. A” (first test) [s]	mean	39.53	35.50	1.45	0.153	62
	sd	12.30	9.86			
	CV	31.11	27.78			
	95% CI	(35.10, 43.97)	(31.94, 39.06)			
After the jump CTT-1 “ver. B” (second test) [s]	mean	34.19	34.50	−0.12	0.908	62
	sd	12.45	8.99			
	CV	35.98	26.05			
	95% CI	(29.75, 38.62)	(31.26, 37.74)			
Before the jump CTT-2 “ver. A” (first test) [s]	mean	72.59	66.91	1.64	0.106	62
	sd	16.56	10.53			
	CV	22.81	15.73			
	95% CI	(66.62, 78.56)	(63.11, 70.70)			
After the jump CTT-2 “ver. B” (second test) [s]	mean	64.69	65.12	−0.26	0.798	62
	sd	15.94	15.23			
	CV	24.64	23.19			
	95% CI	(58.94, 70.43)	(60.20, 71.18)			

CTT, Color Trails Test; sd, standard deviation; CV, coefficient of variation [%]; 95 CI, 95% confidence interval; df, degrees of freedom; *p* significant at *p* < 0.05 level.

**Table 2:** Statistical analysis using the Student’s *t*-test for dependent samples of the significance of differences in mean CTT-1 and CTT-2 test scores before and after the PJ for the EG and CTT-1 and CTT-2 scores in the first and second tests for the CG.

Group	Test	t-Statistic	*p*-Value	df
EG	CTT-1 (pre vs. post)	**2.74**	**0.010**	**31**
	CTT-2 (pre vs. post)	**2.50**	**0.018**	**31**
CG	CTT-1 (pre vs. post)	1.18	0.247	31
	CTT-2 (pre vs. post)	0.73	0.473	31

df, degrees of freedom; bold *p*, significant at *p* < 0.05 level.

## Data Availability

Raw data presented in the study are not publicly available to preserve individuals’ privacy under the European General Data Protection Regulation. To access the data, the first author should be contacted.
